# Management of patient with Fusobacterim nucletum related pleural empyema: intrapleural antibiotic therapy can be considered for salvage therapy

**DOI:** 10.1186/s12879-024-09582-9

**Published:** 2024-07-06

**Authors:** Jingjing Wang, Jing Li, Zhanfei Sun, Shu Zhang, Li Ma, Xiaomei Liu, Xiaoyun Yang, Junqiang Ai, YuanSong, Liang Sun, Xuewen Li, Tao He, Yueyong Xiao, Hongmei Gao, Fei Yuan

**Affiliations:** 1 Department of Intensive Care Unit, Tianjin First Center Hospital, No.24 Fukang Street of Nankai District, Tianjin, 300152 China; 2 Department of MRI, Afliated Hospital, Logistics University of Chinese People’s Armed Police Forces, Tianjin, 300162 China; 3 Department of rheumatology, Afliated Hospital, Logistics University of Chinese People’s Armed Police Forces, Tianjin, 300162 China; 4 Department of Anesthesiology, The First Medical Center of Chinese PLA General Hospital, Beijing, 100039 China; 5 Department of Geriatrics, Characteristic Medical Center of Chinese People’s Armed Police Force, Tianjin, China; 6 Department of Radiology, The First Medical Center of Chinese PLA General Hospital, Beijing, 100039 China; 7 Department of Pathology, Characteristic Medical Center of Chinese People’s Armed Police Force, Tianjin, 300162 China

**Keywords:** Fusobacterium nucleatum, Intrapleural therapy, Pleural empyema

## Abstract

Pleural empyema can lead to significant morbidity and mortality despite chest drainage and antibiotic treatment, necessitating novel and minimally invasive interventions. Fusobacterium nucleatum is an obligate anaerobe found in the human oral and gut microbiota. Advances in sequencing and puncture techniques have made it common to detect anaerobic bacteria in empyema cases. In this report, we describe the case of a 65-year-old man with hypertension who presented with a left-sided encapsulated pleural effusion. Initial fluid analysis using metagenomic next-generation sequencing (mNGS) revealed the presence of Fusobacterium nucleatum and Aspergillus chevalieri. Unfortunately, the patient experienced worsening pleural effusion despite drainage and antimicrobial therapy. Ultimately, successful treatment was achieved through intrapleural metronidazole therapy in conjunction with systemic antibiotics. The present case showed that intrapleural antibiotic therapy is a promising measure for pleural empyema.

## Introduction

Pleural infection has long been associated with significant morbidity and mortality, but advancements in treatment have improved outcomes [1]. The Pleural Infection Longitudinal OuTcome (PILOT) study revealed that standard medical therapy, including pleural tube drainage and systemic antibiotics, is unsuccessful in approximately 33.5% of cases [2]. Surgery [3] and thoracoscopy [4] are now recommended for patients who do not respond to standard treatment, while intrapleural fibrinolytic therapy may be considered for those who are not suitable for surgery [5]. Despite these new interventions, there is limited evidence supporting their effectiveness and they can be costly [1]. Therefore, there is a need for further research to improve patient outcomes in cases of empyema.

Fusobacterium nucleatum induced anaerobic pleural infections are often linked to microaspiration of oral and gastric secretions. Due to challenges in isolating and culturing anaerobes, these infections have historically been misdiagnosed [6]. Fusobacterium nucleatum is also associated with various other conditions, such as colorectal carcinoma, inflammatory bowel disease, respiratory infections, and Lemierre’s syndrome [7]. Although rare in immunocompetent individuals, we present a case of an immunocompetent patient with encapsulated pleural effusion caused by a mixed infection of Fusobacterium nucleatum and Aspergillus chevalieri. The patient was successfully treated with intrapleural metronidazole therapy and systemic antimicrobial infusions.

## Case report

A 65-year-old male patient presented to the emergency department with a ten-day history of left-sided chest wall pain accompanied by cough and white sputum. The chest pain was described as dull and exacerbated by coughing. After eight days, the patient developed additional chest pain. His past medical history included hypertension. On admission, the patient had a wheezing face. The patient’s left thoracic cavity was solid to percussion, and the left breath sounds decreased.

**Fig. 1:**
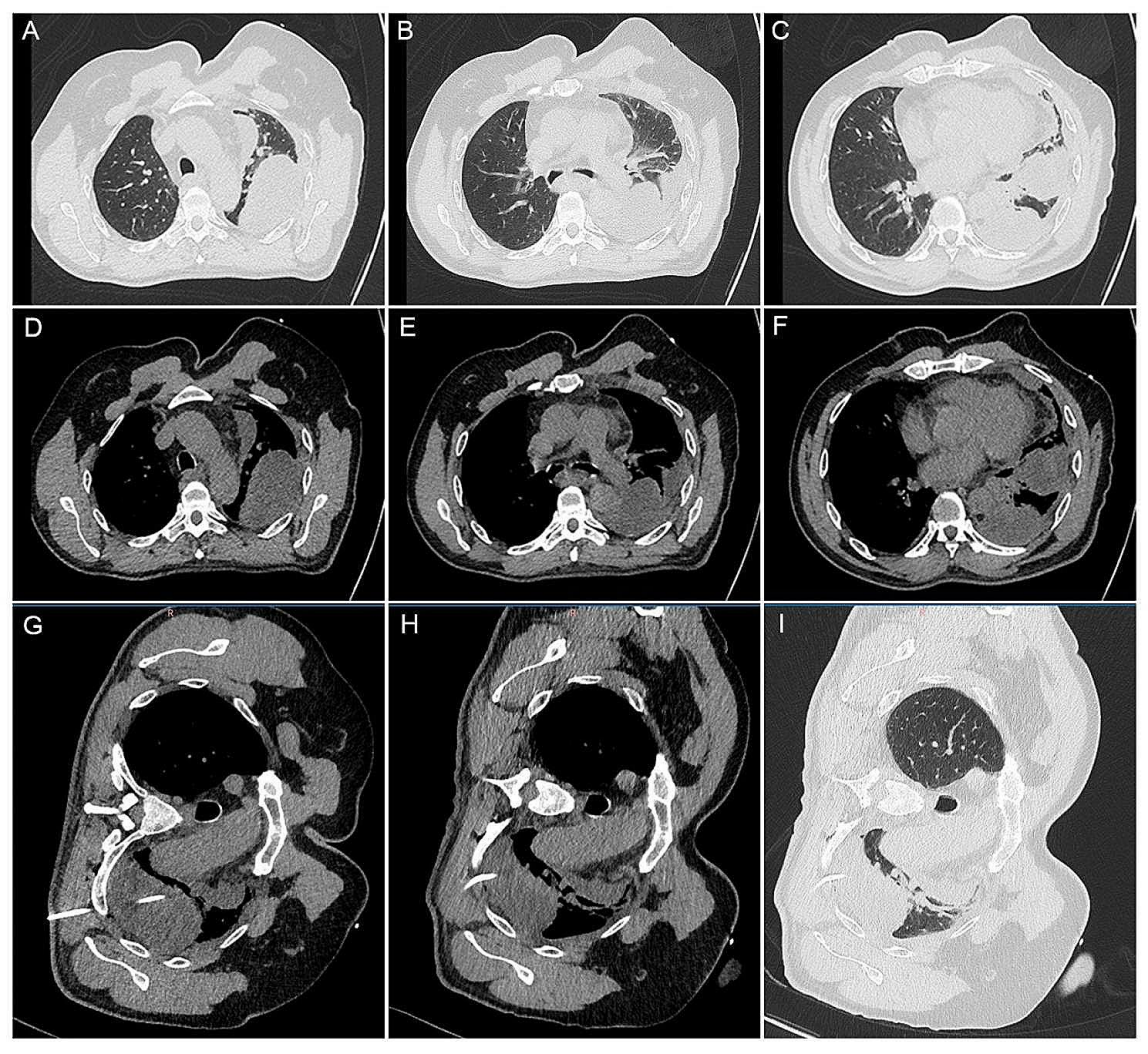
Chest radiological signs of the patient. (**A-F**) Chest CT showed encapsulated pleural effusion on admission. (**G-I**) 8 F pigtail catheter (NNU8LPT, Bard Access Systems, Inc) was placed into left side pleural effusion guided by CT scan

**Fig. 2:**
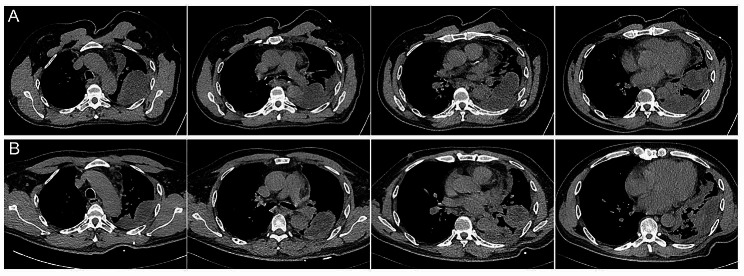
Chest CT scan showed no significant reduction of pleural effusion after chest tube placement and systemic antibiotics treatment for 8 days. (**A**) Chest CT findings on admission. (**B**) Chest CT scan 8 days after chest tube placement and systemic antibiotic therapy

Chest CT revealed a left-sided encapsulated pleural effusion (Fig. 1A and F). Laboratory tests showed leukocytosis of 13.64 × 10^9^/L, slightly elevated PCT of 0.12ng/mL, normal serum myocardial enzymes, elevated D-Dimer of 720 µg/L, and arterial hypoxemia of 64mmHg. The patient underwent thoracentesis with chest tube placement guided by CT (Fig. 1G and I), draining purulent yellow effusion. Further examination of the pleural effusion based on the Light criterion indicated empyema (Pleural fluid characteristics: PH 8.0, Specific gravity 1.033, Protein 50.1 g/L, LDH 1134U/L, number of nucleated cell 170 × 10^6^/L, number of Polymorphonuclear leukocyte 75 × 10^6^/L). Routine pleural culture and acid-fast staining were negative. Analysis of the pleural fluid using mNGS revealed a mixed infection of Fusobacterium nucleatum (2041 Reads per ten million) and Aspergillus chevalieri (Mangin) Thom et Church (4 Reads per ten million). The patient was treated with piperacillin/tazobactam and moxifloxacin for 8 days. A follow-up chest CT showed an increase in the size of the left-sided pleural effusion (Fig. 2) with persistently high inflammatory indicators (The count of white cell was 13.21 × 10^9^/L, PCT 1.05ng/mL) (Table 1).

**Fig. 3:**
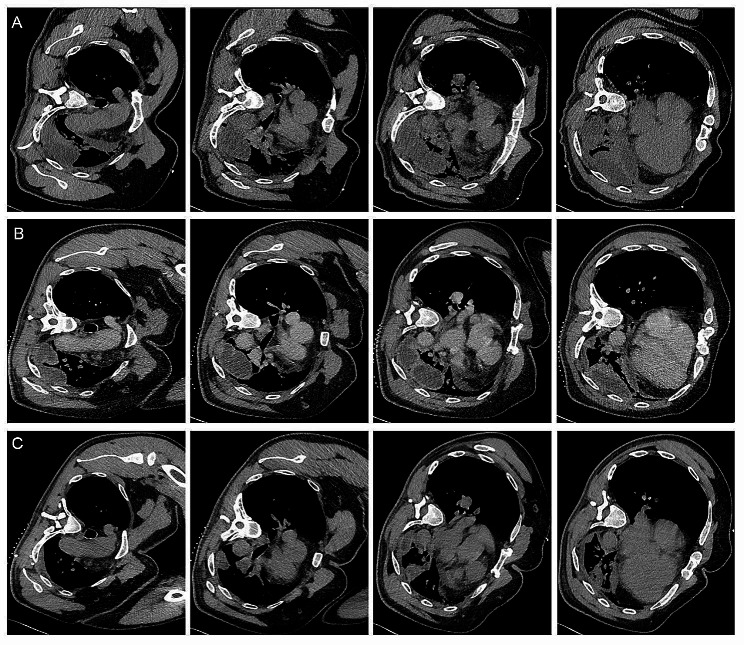
Temporal changes on chest CT imaging during the course of disease. (**A**) Chest CT scan before the first injection of metronidazole and sodium chloride into pleural effusion. (**B**) Imaging changes 7 days after the first intrathoracic injection on chest CT scan. (**C**) Imaging changes 14 days after the first intrathoracic injection on chest CT scan

Subsequently, the patient underwent intrapleural injection therapy of metronidazole and sodium chloride for 3 courses every 7 days, along with systemic intravenous administration of cefoperazone sodium and sulbactam sodium for a 12-day course. The intrapleural injection procedure was as follows. In this case, metronidazole sodium chloride (metronidazole 0.5 g/100mL, sodium chloride 0.8 g/mL, Baxter Medical Co, Shanghai) was chosen. Under CT-guidance, puncture needles were placed individually into the pleural cavities separating the packages and 20–30 ml of pleural fluid was withdrawn, followed by injection of an equal volume of metronidazole sodium chloride solution. Chest CT was reviewed after injection, suggesting a volume effect of the injected metronidazole solution. Finally the puncture needle was removed. Inflammatory markers normalized, with white cell count decreasing to 8.09 × 10^9^/L and PCT decreasing to 0.05ng/mL (Table 1). The patient was discharged in improved condition (Fig. 3) and prescribed oral nemonoxacin for a 14-day course. At the one-month follow-up, the patient was symptom-free and did not undergo the scheduled imaging.

**Table 1:** Inflammatory markers before and after metronidazole injection

Index	before	After
WBC	13.21 × 10^9^/L	8.09 × 10^9^/L
PCT	1.05ng/mL	0.05ng/mL

## Discussion

Pleural infection is a serious disease that commonly occurs as a secondary infection of severe pneumonia. There is a growing recognition of primary pleural infection without parenchymal involvement. Standard measures against pleural infection include appropriate antimicrobial therapy and chest-tube drainage. New interventions such as thoracoscopy to clear the infected space, intrapleural fibrinolytic therapy, high-volume pleural irrigation with saline/antiseptic solution, and repeated thoracentesis have been introduced [1]. Despite these advancements, pleural infection remains life-threatening, with approximately 15% of patients requiring emergent treatment [8, 9]. The disease poses a significant medical burden, often necessitating prolonged hospitalization. Sometimes surgery can’t be performed because of comorbidities. Therefore, there is a need for new minimally invasive treatment methods. This article presents a case of multiple intrapleural antibiotic therapy under CT guidance in a patient with primary pleural infection.

Accurate etiology and timely anti-infection therapy are essential for improving the prognosis of patients with pleural infections. The most common organisms found in pleural fluid samples are gram-positive cocci, specifically S. pneumoniae, followed by aerobic gram-negative bacilli and anaerobic bacteria [1]. The use of next- generation sequencing has revealed that up to 70% of pleural fluid samples contain anaerobic bacteria [10]. In this particular case, pleural effusion was identified using CT imaging due to regional and multilocular effusion. Fusobacterium, an anaerobic bacteria, is responsible for 0.6–3.5 cases per 1 million population [11] and is often associated with microaspiration of oral secretions and gastric content. These infections are commonly seen in individuals with neurological conditions affecting swallowing, immunocompromised patients, or those at high risk of aspiration [1]. The patient in this case had normal white blood cell count and CD_4_^+^/CD_8_^+^ T cell ratio (CD4^+^/CD8^+^ 2.07), indicating normal immune function, and did not have any neurological comorbidities. Risk factors for anaerobic empyema development include bacterial pneumonia, surgery or chest trauma, esophageal perforation, thoracentesis, subdiaphragmatic infection, spontaneous pneumothorax, bacteremia, and tobacco or alcohol use [10, 12]. In this case, the patient’s only risk factor was alcohol abuse.

Appropriate antimicrobial therapy and chest-tube drainage are essential components of treating pleural infections. The patient in this case received immediate chest-tube drainage and was prescribed piperacillin/tazobactam with moxifloxacin as empirical antibiotic therapy. Both β-lactam and moxifloxacin are believed to penetrate the pleura effectively, as indicated by animal studies demonstrating higher concentrations in pleural fluid [13]. However, the translation of these findings to human patients remains uncertain [14]. The therapeutic response in this case was suboptimal, possibly due to the fibropurulent stage, which may reduce the effectiveness of antibiotic treatment. Furthermore, inadequate drug concentrations and limited systemic antibiotic efficacy in infected tissues can contribute to the development of antibiotic resistance. Thus, intravenous antibiotics alone may not be effective in this case. Intrathoracic local administration increases local tissue drug concentrations. As an adjunct to intravenous anti-infective therapy, intrathoracic antibiotic injection improves bacterial clearance and promotes absorption in the abscessed chest.

## Conclusion

Ultimately, the specific microenvironments in infected tissues exacerbate antibiotic resistance by hindering the effectiveness of systemic antibiotic treatments and decreasing drug levels at the site of infection, increasing the risk of recurrent infections. To address the limitations of systemic drug administration, utilizing polymeric carriers for encapsulation has been demonstrated to improve antimicrobial effectiveness, penetration, and retention at the infection site. Although there is limited evidence on intrapleural antibiotics, mainly consisting of case reports or case series [15, 16], the encouraging outcomes indicate that intrapleural antibiotics hold promise for future research. Pleural injection can be suggested when thoracocentesis with systemic antiobiotherapy have failed and when surgery can’t be performed because of comorbidities or because of patient refusal.

## Data Availability

Data is provided within the manuscript or supplementary information files.
